# Lotka–Volterra dynamics kills the Red Queen: population size fluctuations and associated stochasticity dramatically change host-parasite coevolution

**DOI:** 10.1186/1471-2148-13-254

**Published:** 2013-11-19

**Authors:** Chaitanya S Gokhale, Andrei Papkou, Arne Traulsen, Hinrich Schulenburg

**Affiliations:** 1 Evolutionary Theory Group, Max Planck Institute for Evolutionary Biology, August Thienemann Str-2, 24306, Plön, Germany; 2 Department of Evolutionary Ecology and Genetics, Christian-Albrechts-University of Kiel, 24098, Kiel, Germany

**Keywords:** Host-parasite coevolution, Red Queen hypothesis, Lotka-Volterra dynamics, Genetic drift, Population bottleneck

## Abstract

**Background:**

Host-parasite coevolution is generally believed to follow Red Queen dynamics consisting of ongoing oscillations in the frequencies of interacting host and parasite alleles. This belief is founded on previous theoretical work, which assumes infinite or constant population size. To what extent are such sustained oscillations realistic?

**Results:**

Here, we use a related mathematical modeling approach to demonstrate that ongoing Red Queen dynamics is unlikely. In fact, they collapse rapidly when two critical pieces of realism are acknowledged: (i) population size fluctuations, caused by the antagonism of the interaction in concordance with the Lotka-Volterra relationship; and (ii) stochasticity, acting in any finite population. Together, these two factors cause fast allele fixation. Fixation is not restricted to common alleles, as expected from drift, but also seen for originally rare alleles under a wide parameter space, potentially facilitating spread of novel variants.

**Conclusion:**

Our results call for a paradigm shift in our understanding of host-parasite coevolution, strongly suggesting that these are driven by recurrent selective sweeps rather than continuous allele oscillations.

## Background

The Red Queen from Lewis Carroll’s tale ‘Through the looking glass’ is commonly used as a metaphor for selection-induced rapid evolution [1-3]. It is based on the observation that persistence in an environment with changing selective constraints requires ongoing adaptations to the encountered challenges [4]. Host-parasite coevolution with antagonistic and inter-dependent interactions represents one of the role models for such rapid evolutionary change [5,6]. For instance, an increase in host resistance reduces parasite fitness, thus immediately favoring parasite varieties with altered virulence and/or immune-evasion mechanisms. In turn, a novel parasite attack mechanism decreases host fitness, thus favoring host varieties with new counter-defenses. If the interaction persists, then it will lead to continuous parasite adaptations and host counter-adaptations. The rapid evolutionary dynamics associated with these interactions is very well documented in the literature, ranging from field studies on rabbits and their myxoma viruses [7], snails and their trematode parasites [8], *Daphnia magna* waterfleas and their bacterial parasites [9] to laboratory-based coevolution experiments between bacteria and their phages [10-12], the nematode *Caenorhabditis elegans* and bacterial parasites [13,14], or the red flour beetle *Tribolium castaneum* and its microsporidian parasite [15,16].

It is thus widely accepted that these interactions evolve fast and continuously. Yet, to date, the exact underlying selection dynamics are not always well understood. These dynamics can generally be influenced by metapopulation structure and environmental variation [17,18]. Within a particular population and specific environmental context, two main alternatives are thought to be of prime importance: recurrent selective sweeps and negative frequency-dependent selection [5,6,19-21]. Both alternatives are consistent with the above original definition of the Red Queen hypothesis by Van Valen [4], whereas, curiously, only the second alternative is referred to as Red Queen dynamics [5,6,20]. The two alternatives are closely related because both assume a selective advantage of a rare genotype, for example a novel host resistance variant. However, they differ fundamentally in the way in which the new variant originates and spreads within the population. The concept of recurrent selective sweeps (often termed *arms race dynamics*) consists of two steps: the *de novo* appearance of a beneficial allele (e.g., by mutation or immigration) and its subsequent spread through the population to fixation (i.e., the selective sweep). These sweeps occur repeatedly in host and parasite populations, usually each time with a new beneficial allele. They may only lead to fast changes in absolute time if at least one of the following factors applies: new alleles arise frequently, new alleles become immediately visible and thus selectable at the phenotypic level, the new alleles provide a high selective advantage, and/or the organisms have short generation times. This situation is best met in bacteria-phage interactions, which are usually characterized by large population sizes (i.e., high likelihood of the occurrence of favorable mutations), short generation times, and haploid genomes (i.e., new mutations are immediately expressed phenotypically) [11,22-24] (but see also [25]).

In contrast, the dynamics for multicellular host systems are traditionally viewed to be determined by negative frequency-dependent selection leading to sustained oscillations of the same alleles (i.e., Red Queen dynamics [6,20]), but not to the fixation of single alleles. In this case, standing genetic variation is required, because the population sizes for these hosts are usually comparatively small, their generation times comparatively long, and their genomes diploid. As a consequence, recurrent selective sweeps are commonly thought to be rather slow in these systems. Instead, if standing genetic variation is available, then negative frequency-dependent selection can produce fast and continuous allele frequency changes even in these host systems. Such negative frequency-dependent dynamics seem to be present in some multicellular host systems, including the freshwater snail *Potamopyrgus antipodarum*[8,26] and the waterflea *Daphnia magna*[9].

Numerous theoretical models have been developed to study the underlying selection dynamics. Interestingly, the current models typically focus on evolutionary change (i.e., the rate of change in host and parasite allele frequencies in response to the type of interaction). These approaches have thus largely neglected ecological dynamics, which can have a huge impact on the evolutionary process. Population size fluctuations deserve particular attention in this context, because they are induced by reciprocal selection among the antagonists and, therefore, represent an inherent property of host-parasite coevolution - irrespective of additional environmental variation [7,10,27-30]. Since selection is reciprocal, population size fluctuations should be coupled between the antagonists, and generally follow Lotka-Volterra dynamics [31,32]. Such demographic variations have the potential to affect the dynamics of host-parasite allele frequency changes by introducing two important effects. Firstly, the rising and falling population sizes produce bottlenecks where selection favours a particular allele. The favored allele may thus reach comparatively high frequencies during the bottleneck, possibly enhancing its spread in the subsequently expanding population. Secondly, the elevated stochasticity during the bottleneck may lead to a further increase and thus spread of the favored allele.

In this manuscript, we aim at understanding in how far Lotka-Volterra population size fluctuations and the associated stochastic effects influence the dynamics of allele frequency changes during host-parasite coevolution. While several previous theoretical models have applied the Lotka-Volterra dynamics to host-parasite coevolution (e.g., [33-37]), their influence on the evolutionary dynamics has not yet been systematically explored by comparison with a model with constant population size. Similarly, stochastic effects during host-parasite coevolution have only been considered in a few theoretical studies (e.g., [38,39]), yet, to our knowledge, with a single exception [40] under constant population size and not in combination with Lotka-Volterra dynamics. Hence, while the previous studies have independently utilised stochastic effects or Lotka-Volterra dynamics, a systematic analysis of the consequences of each of these factors, either alone or in combination, is as yet missing - in spite of their potential importance. The novelty of our study lies in bringing together these two aspects and comparing their influence to the traditional model, in which Lotka-Volterra dynamics and stochastic effects are excluded. More specifically, we here use the standard matching-alleles host-parasite interaction model to assess allele frequency dynamics in the presence versus absence of Lotka-Volterra oscillations for a stochastic versus an analogous deterministic model.

## Methods

Based on the Lotka-Volterra equations [31,32], we address the population dynamics of interacting hosts and parasites. The host corresponds to the prey in the original model, and the parasite to the predator. The host consumes a (constant) food supply *F* and reproduces at rate *c*_1_. Parasites infect hosts at rate *c*_2_, leading to elimination of a host and generation of an additional parasite. Parasites die at rate *c*_3_. The number of host and parasite individuals are given by *H* and *P*. In a stochastic system these interactions can be defined by the following reactions [41,42],

(1) $$\begin{array}{lll} F+H\;\; & \overset{c_{1}}{\to }\;\;H+H\; & \;\\ H+P\;\; & \overset{c_{2}}{\to }\;\;P+P\;\\ \;P\;\;\overset{c_{3}}{\to }\;\;0.\; & \; \end{array}$$

Usage of these specific reactions facilitates tracking of each unit of the interacting antagonists and, thus, it allows a more precise characterization of the resulting dynamics. These reactions can also be used directly for exact stochastic simulations based on the Gillespie algorithm. They further provide a microscopic dynamics from which the deterministic Lotka-Volterra equations emerge in the limit of infinite population size [42],

(2) $$\begin{array}{ll} \dot{H}\;\;=\;\;c_{1}F\;H-c_{2}H\;P\; & \;\\ \dot{P}\;\;=\;\;c_{2}H\;P-c_{3}\mathrm{P.}\; \end{array}$$

Host-parasite coevolution is modeled using the standard matching alleles model [6]. For this, we define two host and two parasite types, *H*_1_ and *H*_2_ for the host and *P*_1_ and *P*_2_ for the parasite. This is equivalent to a haploid system with two antagonists, each of which possesses two alleles at a single locus. The interaction according to the matching alleles model is described with the following six reactions,

(3) $$\begin{array}{ll} \;H_{1}\;\;\overset{ã}{\to }\;\;H_{1}+H_{1}\; & \;\\ \;H_{2}\;\;\overset{ã}{\to }\;\;H_{2}+H_{2}\; & \;\\ H_{1}+P_{1}\;\;\overset{\tilde{b}}{\to }\;\;P_{1}+P_{1}\; & \;\\ H_{2}+P_{2}\;\;\overset{\tilde{b}}{\to }\;\;P_{2}+P_{2}\; & \;\\ \;P_{1}\;\;\overset{\tilde{c}}{\to }\;\;0\; & \;\\ \;P_{2}\;\;\overset{\tilde{c}}{\to }\;\;0.\; \end{array}$$

In the matching alleles model, the interactions between alternate hosts and parasites (*H*_1_, *P*_2_ and *H*_2_, *P*_1_) are without consequence and thus do not appear here. While the absence of these interactions is the standard assumption in the matching alleles model, allowing a small amount of these interactions does not change our results qualitatively (see Appendix). In the limit of infinite population size [42], we obtain a set of four coupled nonlinear differential equations,

(4) $$\begin{array}{ll} \dot{h_{1}}\;\;=\;\;h_{1}(a-bp_{1})\; & \;\\ \dot{h_{2}}\;\;=\;\;h_{2}(a-bp_{2})\; & \;\\ \dot{p_{1}}\;\;=\;\;p_{1}(bh_{1}-c)\; & \;\\ \dot{p_{2}}\;\;=\;\;p_{2}(bh_{2}-c),\; \end{array}$$

where the frequencies of *H*_*i*_ and *P*_*i*_ are given by *h*_*i*_ and *p*_*i*_. The above equations consider interdependence of host and parasite demographies, allowing population sizes to vary in response to the interaction with the antagonist, consistent with the Lotka-Volterra model. The precise nature of the resulting oscillations in population size is determined by the parameters, most importantly by *b*.

As we are interested in the effects of population size variation induced by the Lotka-Volterra equations, we have to compare this to a scenario in which the population size is constant. Such constant population size models are common, e.g. the Wright-Fisher model or the Moran process. However, microscopically these models are distinct from the Lotka-Volterra equations considered above. Therefore, we used the above approach and enforced constant population size by resetting host and parasite population sizes to their initial values after every generation (*N*_avg_ transition events, see Appendix), while relative allele frequencies were maintained. The dynamics were subsequently assessed for different average population sizes. To ensure comparability of allele frequency fluctuations across population sizes and evolutionary models, we rescaled the interaction parameters with *N*_avg_ for the deterministic analogues of the considered stochastic scenarios (Appendix).

## Results

**Figure 1:**
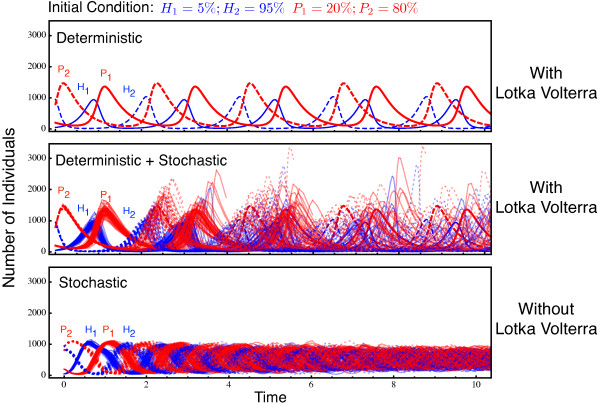
**Example of allele frequency dynamics with and without Lotka-Volterra population size fluctuations.** Top: Lines show the deterministic Lotka-Volterra dynamics, as often considered in theoretical studies, cf. Eqs. (4). Middle: When stochasticity is included (thin lines show the results of 50 individual stochastic Gillespie simulations), then simulations may initially produce allele oscillations as above and below. However, alleles usually spread to fixation (or go extinct) at a much faster rate. Bottom: Dynamics without Lotka-Volterra cycles, fixing the average population size of both species to *N*_avg_=1000 by resetting it after every *N*_avg_ reactions, while maintaining the ratio between the alleles. The 50 individual stochastic simulations now only rarely reach fixation. The figure illustrates the scenario where the rare host allele (*H*_1_) is more likely to reach fixation than the frequent host allele (see Figure 3). This fixation probability decreases with increasing initial frequency (cf. Figure 3). The simulation parameters are *a*=5, *c*=2.5, *b*=10/*N*_avg_=0.01 with *H*_1_=5*%*, *H*_2_=95*%*, *P*_1_=20*%*, *P*_2_=80*%* as initial condition.

Host-parasite coevolutionary dynamics are analyzed in the presence and absence of Lotka-Volterra dynamics. Figure 1 illustrates an exemplary result. All models initially produce oscillatory allele frequency changes, but only with Lotka-Volterra dynamics are these accompanied by changes of population size. As a consequence, changes in allele numbers are also more pronounced (top versus bottom in Figure 1). As the deterministic model allows for arbitrary small frequencies of each type, it formally never leads to allele fixation and thus produces continuous oscillations. In contrast, the corresponding stochastic models have absorbing states, making fixation possible. Interestingly, allele fixation appears to be substantially faster in the stochastic model that includes Lotka-Volterra fluctuations (top versus bottom panels, Figure 1). As such, it seems that these conditions favor rapid termination of the Red Queen oscillations.

**Figure 2:**
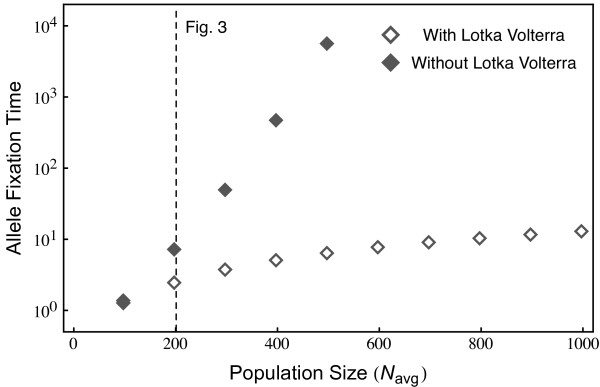
**The time until fixation of one allele, either host or parasite, is shown in dependence of the initial population size*N***_***avg***_**.** Including Lotka-Volterra fluctuations, the fixation time is only weakly affected by increases in *N*_*avg*_. Excluding Lotka-Volterra cycles maintains allele frequency oscillations, leading to an exponential increase in fixation time as *N*_*avg*_ increases. For all simulations the initial condition were *H*_1_=5*%*, *H*_2_=95*%*, *P*_1_=20*%*, *P*_2_=80*%* of the *N*_*avg*_, and the parameters *μ*=5, *c*=2.5, *b*=10/*N*_*avg*_ with averages over 10^6^ realizations). The vertical dotted line shows the population size employed in Figure 3.

We next analyze the impact of the average population size on this pattern. In the following, we focus on the time until one of the alleles from either of the antagonists has reached fixation in order to compare evolutionary rates across population sizes and models. In general, Lotka-Volterra dynamics cause a substantial increase in allele fixation rate (Figure 2). Interestingly, in this case, allele fixation rates depend only weakly on average population size. Figure 1 suggests that this is because allele frequencies can become very small during the Lotka-Volterra demographic fluctuations. In contrast, average population size has a much stronger effect when it is artificially kept constant. Here, the time until allele fixation increases exponentially with increasing population size (Figure 2).

**Figure 3:**
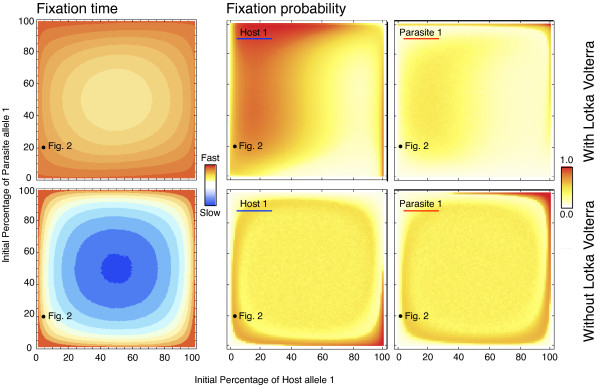
**The influence of initial allele frequency on fixation time and probability for the stochastic models.** For an *N*_*avg*_=200, we plot the time until any of the four alleles goes to fixation (left column) and the probability of fixation of one of the host and parasite alleles for all possible initial conditions (middle and right columns) (averages over 10^6^ realizations). Lotka-Volterra fluctuations lead to substantially faster allele fixations (top left panel) and high fixation probability for the host allele across a wide range of initial conditions (top middle panel). The simulations were always stopped when either one of the host or one of the parasite alleles reached fixation. Thus, the sum of the fixation probabilities of all alleles sums up to 1. The specific initial conditions used in Figure 2 are indicated.

**Figure 4:**
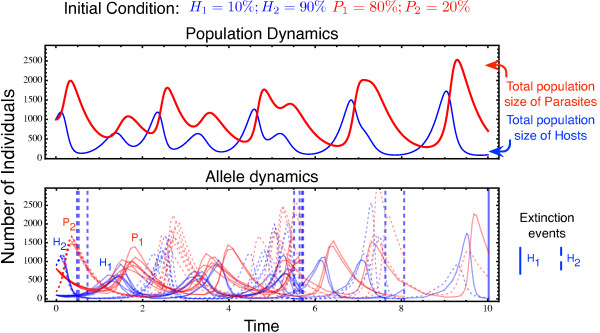
**Detailed dynamics explaining the seemingly counterintuitive result of the high fixation probability of Host allele 1 in spite of the high prevalence of Parasite allele 1 (in Figure**3**top middle panel) due to the inclusion of Lotka-Volterra dynamics.** In this figure we depict the dynamics that occur at the initial conditions with a low *H*_1_ frequency and a high *P*_1_ frequency. In this particular case, the low initial frequency of *H*_1_ means that *H*_2_ is initially common, which in turn favours *P*_2_. This parasite allele thus rapidly increases in frequency, subsequently causing highly pronounced *H*_2_ and *P*_2_ frequency oscillations that show larger amplitudes than the interacting *H*_1_ and *P*_1_ alleles. If low *H*_2_ frequencies coincide with a Lotka-Volterra bottleneck in the hosts, then the associated stochastic effects lead to a higher likelihood of *H*_2_ going extinct, resulting in an overall higher fixation probability of *H*_1_. The top panel shows the average population dynamics, whereas the bottom panel shows the frequency changes for the indicated host and parasite alleles across the ten independent simulations. The vertical lines in the bottom panel denote the time points where the simulation is terminated due to a loss of an allele. Out of the 10 simulation runs 9 are stopped due to the allele *H*_2_ going extinct and only one due to *H*_1_ going extinct. The interaction parameters are *a*=5, *c*=2.5, *b*=10/*N*_avg_=0.01.

Figure 2 explores the time to fixation of any of the alleles in either the host or the parasite using a specific combination of initial allele frequencies (i.e., the rare host allele is present at 5%, the common at 95%, whereas the parasite alleles are at 20% and 80% respectively). How does this depend on the initial allele frequencies in both antagonists? For instance, the selective advantage of a rare allele is not only the result of its own frequency, but also determined by the abundance of the corresponding allele in the antagonist. Allele fixation rates were thus explored as a function of initial allele frequencies in the two antagonists. Most impressively, Lotka-Volterra fluctuations cause much faster allele fixation under almost all initial conditions (Figure 3, left column, top versus bottom panel). The detailed analysis then suggests that, in case of Lotka-Volterra fluctuations, host alleles can have a high fixation probability even if initially rare (Figure 3, middle panel in top row). This is true across a relatively wide distribution of initial frequencies for the corresponding parasite allele. Interestingly, it even applies when the corresponding parasite allele has high initial frequencies (Figure 3, top left corner in top middle panel). This counterintuitive result can be explained by consideration of the dynamics that ensue from these initial conditions. In this particular case, the low initial frequency of host allele 1 means that host allele 2 is initially common, whereas the high initial frequency of parasite allele 1 means that parasite allele 2 is rare. High host allele 2 abundance then specifically favors parasite allele 2, which rapidly increases in frequency. The unexpected consequence of these starting conditions is that these two interacting alleles subsequently engage in highly pronounced frequency oscillations that show larger amplitudes than those observed for host and parasite alleles 1 (Figure 4). If during these oscillations low allele 2 frequencies coincide with a Lotka-Volterra bottleneck and associated stochastic effects, then host allele 2 has a very high likelihood of going extinct, resulting in fixation of host allele 1 (see Figure 4).

The results also highlight that the dynamics are usually determined by fixation of one of the host alleles (red colour is mainly found in middle rather than right panel of the top row of Figure 3). Note that the simulations are stopped as soon as either one of the host or one of the parasite alleles reaches fixation and, thus, the fixation probabilities of both host as well as both parasite alleles sum up to one. In our case, fixation of the host allele is more likely than fixation of the parasite allele because for our parameter combination and initial condition, it is usually the host that first experiences a Lotka-Volterra bottleneck and consequentially a drop in the frequency of one of the alleles (see also Figure 4). Nevertheless, if both the parasite and corresponding host allele are common, then it is the parasite allele that has a high probability of fixation (Figure 3, top right).

The overall pattern looks different in the absence of Lotka-Volterra fluctuations (Figure 3, bottom row). Host allele fixation probability increases with its own high initial frequency and, at the same time, low initial abundance of the corresponding parasite allele (Figure 3 middle panel in bottom row). Parasite allele fixation is enhanced when both parasite and corresponding host alleles are initially common (Figure 3 bottom right). Under these conditions, fixation probabilities of both host and parasite alleles are almost identical at initially intermediate frequencies, most likely due to negative frequency dependent selection, as illustrated in Figure 1.

## Discussion

Population size fluctuations represent an inherent property of host-parasite interactions. Unequivocal evidence for such interaction-dependent demographic variations was obtained from controlled host-parasite mesocosm experiments, for example with *E. coli* and its phage [10,30], *Hydra* hosts and its *Hydramoeba* parasite [43], house fly and its parasitic wasps [44,45], or azuki bean weevil *Callosobruchus chinensis* and its parasitoid *Heteropilus prosopidis*[46]. Similar observations were made under field conditions, for example for rabbits and their myxoma viruses [7], or red grouse and its nematode parasite [47]. Additional examples are summarized by [28] and [29]. As population size fluctuations produce regular bottlenecks, random genetic drift is likely to influence allele frequencies. Previous theoretical models, developed in a different context, strongly suggest that even large populations are influenced by such stochastic processes [48,49]. More generally, under natural conditions in a finite population, it is difficult to imagine that changes in population size do not affect evolutionary dynamics. Consequently, an in-depth understanding of the evolution of host-parasite interactions should take account of the associated ecological processes based on Lotka-Volterra fluctuations.

Very few previous theoretical models on host-parasite interactions have considered Lotka-Volterra fluctuations [33-37]. These studies usually used a deterministic approach and thus excluded stochastic effects, which are most prominent during bottlenecks. Similarly, only few theoretical studies considered stochastic effects in this context [38,39], yet under constant population size, but not in combination with Lotka-Volterra dynamics. We are aware of only one study that looked at host-parasite coevolution in consideration of Lotka-Volterra interactions and stochasticity [40]. However, this study had a different focus, and thus, it did not include a systematic comparison to models without Lotka-Volterra cycles or without stochasticity. Consequently, the interaction of these two aspects for host-parasite coevolution is so far unexplored. At the same time, their relevance was demonstrated for evolution of only one of the antagonists, namely the parasite. For example, the probability of fixation of a beneficial mutation in a bacterial population was shown to be enhanced by periodical bottlenecks [50-52]. Similar results were obtained in a model that explored the effect of bottlenecks during pathogen transmission [53]. Our study explicitly evaluates the influence of both Lotka-Volterra fluctuations and stochastic effects on the dynamics of host-parasite interactions using a comparison to a model with constant population size and/or absence of stochasticity. As the demographic variations are an inherent property of such antagonistic interactions, their influence should apply across a wide range of environmental conditions and thus be of general relevance for our understanding of host-parasite coevolution.

Based on our approach, we obtained evidence that both Lotka-Volterra fluctuations and associated stochastic effects significantly affect the course and pace of coevolutionary adaptations. In particular, both factors facilitate selective sweeps (i.e., the spread and fixation of an allele). Most impressively, this effect appears to be independent of average population size (Figure 2) and occur at a substantially faster rate (Figure 3, left column). Moreover, allele frequency changes are not exclusively due to drift, which should favor fixation of initially frequent alleles and loss of initially rare alleles. In contrast, our results indicate that initially rare host alleles can spread to fixation across a relatively wide range of conditions (Figure 3, top middle panel). Rare parasite alleles may not necessarily go extinct, but have a certain likelihood of spreading contingent on the frequency of the corresponding host allele (Figure 3, top right panel). Based on these results we propose that selective sweeps rather than oscillatory negative frequency-dependent selection may represent the main driving force during host-parasite coevolution.

Recurrent selective sweeps have been repeatedly suggested to determine coevolutionary dynamics for parasite or host systems with large population sizes such as bacterial hosts or microbial parasites, where novel mutations are frequent and often directly exposed to selection because of a haploid genetic system. If these selective dynamics also apply to multicellular host and parasite systems, then two contrasting effects may be expected on the coevolutionary process. On the one hand, these systems usually have much smaller population sizes, facilitating spread of alleles in spite of the often diploid genetic system. On the other hand, continuous coevolution may become difficult because it is usually assumed that small population size results in a reduced likelihood of the occurrence of advantageous novel mutations [6]. However, the latter assumption may not always be true. It is possible that new alleles become available for example by frequent immigration or a high rate of gene duplication. These processes may further favor the formation of novel genotypes if they act in combination with recombination and/or mutation. Interestingly, the possible impact of gene duplications is usually not addressed in theoretical work on host-parasite coevolution, even though such duplications are known to be common in almost all organisms [54-56] and often affect genes of relevance for the interaction such as virulence genes in parasites [57,58] or immunity genes in animal hosts [54,59,60].

Several additional factors may favor ongoing coevolution. One of these is founded on a more complex genetic architecture underlying host-parasite co-adaptation that consists of several interacting loci across the genome (e.g., [61-64]). In this case, allele fixation at one locus may still permit maintenance of variation at other loci, which could then mediate evolutionary responses to the antagonist. Yet another possibility may depend on metapopulation structure, consisting of coevolutionary hot and cold spots and migration among demes, as evidenced for flax and its parasitic rust fungus [65] or the above mentioned snail-trematode interaction [17]. Such an interconnected network could then maintain allelic diversity across the entire metapopulation, even if alleles become temporarily fixed within single demes. Moreover, environmental gradients or perturbations are known to influence host-parasite coevolutionary dynamics [66]. They could similarly prevent loss of alleles, even if the coevolutionary interaction itself would specifically favour only one of the alleles. Obviously, the above processes act in combination with each other in natural systems. Therefore, it is indeed conceivable that recurrent selective sweeps shape long-lasting coevolutionary dynamics even in multicellular host systems.

## Conclusion

In conclusion, decades of empirical efforts have tried to demonstrate the presence of Red Queen dynamics during host-parasite coevolution. This has led to most ingenious experiments which repeatedly and independently confirmed negative frequency dependence as a driving force [8,9,26,67,68] and such a trend continues to date [21,69]. These studies yielded impressive evidence that parasite abundance typically increases first and, once the host evolves a defense mechanism, it decreases again. However, sustained allele frequency oscillations of a particular allele, as predicted by numerous theoretical models assuming constant population size in the absence of any stochastic effects, have not been reported. We here propose that Lotka-Volterra population size fluctuations and the associated stochastic effects represent an inherent property of host-parasite interactions that can lead to rapid fixation of alleles, even those initially rare, thus preventing sustained oscillations. Consequently, Lotka-Volterra population size fluctuations have the potential to stop the Red Queen - unless novel variants are introduced into the population and/or additional selective constraints maintain allelic diversity. In retrospect, our findings may not be entirely unexpected. However, to date, they have not yet been directly demonstrated using a systematic analysis approach, as implemented here. More importantly, they are generally neglected in the numerous current empirical studies on the topic, in spite of their potential importance. They clearly deserve specific attention in future theoretical and empirical work aimed at an improved understanding of host-parasite coevolutionary dynamics.

## Appendix

### Relating stochastic and deterministic dynamics

Stochastic models are often developed starting from deterministic formulations [42]. Since the same deterministic formulation can be the limiting case of many individual based models, this procedure may be problematic. Instead, beginning from a stochastic, individual based description and then calculating the deterministic analogue will provide only a single direct link between the two approaches and allows for their direct comparison.

We consider a haploid system involving two antagonistic pairs, two alleles in hosts and parasites each. Firstly, all possible changes are written in the form of simple chemical reactions. In our particular case we have eight such possible reactions. We denote the two hosts and the two parasites by *H*_1_ and *H*_2_ and *P*_1_ and *P*_2_ respectively. Thus we have,

(5) $$\begin{array}{ll} \;H_{1}\;\;\overset{\tilde{\mu }}{\to }\;\;H_{1}+H_{1}\; & \;\\ \;H_{2}\;\;\overset{\tilde{\mu }}{\to }\;\;H_{2}+H_{2}\; & \;\\ H_{1}+P_{1}\;\;\overset{\tilde{b}}{\to }\;\;P_{1}+P_{1}\;\\ H_{2}+P_{2}\;\;\overset{\tilde{b}}{\to }\;\;P_{2}+P_{2}\; & \;\\ \;P_{1}\;\;\overset{\tilde{c}}{\to }\;\;0\; & \;\\ \;P_{2}\;\;\overset{\tilde{c}}{\to }\;\;0.\; & \; \end{array}$$

For instance, a parasite 2 individual dies with the rate $\tilde{c}$. From these rate reactions, we obtain the transition rates of the system. Depending on the number of individuals of the different types namely $n=\{n_{H_{1}},n_{H_{2}},n_{P_{1}},n_{P_{2}}\}$, we write the rates as,

(6) $$\begin{array}{ll} \;T(n_{H_{1}}+1,n_{H_{2}},n_{P_{1}},n_{P_{2}}|n)=\tilde{\mu }\frac{n_{H_{1}}}{N_{\text{avg}}}\; & \;\\ \;T(n_{H_{1}},n_{H_{2}}+1,n_{P_{1}},n_{P_{2}}|n)=\tilde{\mu }\frac{n_{H_{2}}}{N_{\text{avg}}}\; & \;\\ T(n_{H_{1}}-1,n_{H_{2}},n_{P_{1}}+1,n_{P_{2}}|n)=2\tilde{b}\frac{n_{H_{1}}}{N_{\text{avg}}}\frac{n_{P_{1}}}{N_{\text{avg}}}\;\\ T(n_{H_{1}},n_{H_{2}}-1,n_{P_{1}},n_{P_{2}}+1|n)=2\tilde{b}\frac{n_{H_{2}}}{N_{\text{avg}}}\frac{n_{P_{2}}}{N_{\text{avg}}}\; & \;\\ \;T(n_{H_{1}},n_{H_{2}},n_{P_{1}}-1,n_{P_{2}}|n)=\tilde{c}\frac{n_{P_{1}}}{N_{\text{avg}}}\; & \;\\ \;T(n_{H_{1}},n_{H_{2}},n_{P_{1}},n_{P_{2}}-1|n)=\tilde{c}\frac{n_{P_{2}}}{N_{\text{avg}}}\; & \; \end{array}$$

where the reaction rates, have been corrected by each reactions combinatorial possibility [70,71] and *N*_avg_ is the average population size which we consider to be the same for the hosts as well as the parasites (the difference in the average population size can be interpreted as the ratio between the growth rate of hosts and the death rate of parasites). Using these rates, we can write down deterministic differential equations for the change in the average number of a certain type, e.g.

(7) $$\begin{array}{l} \frac{d\langle n_{H_{1}}\rangle }{\text{dt}}=\tilde{\mu }\frac{n_{H_{1}}}{N_{\text{avg}}}-2\tilde{b}\frac{n_{H_{1}}}{N_{\text{avg}}}\frac{n_{P_{1}}}{N_{\text{avg}}}. \end{array}$$

Introducing rescaled reaction rates, $\mu =\frac{\tilde{\mu }}{N_{\text{avg}}}$, $b=2\frac{{\tilde{b}}_{}}{N_{\text{avg}}}$ and $c=\frac{\tilde{c}}{N_{\text{avg}}}$, we obtain

(8) $$\begin{array}{l} \frac{d\langle n_{H_{1}}\rangle }{\text{dt}N_{\text{avg}}}=\mu \langle \frac{n_{H_{1}}}{N_{\text{avg}}}\rangle -b\langle \frac{n_{H_{1}}}{N_{\text{avg}}}\frac{n_{P_{1}}}{N_{\text{avg}}}\rangle . \end{array}$$

In the limit of a large population size we recover the mean field approximation or the population level model [71],

(9) $$\begin{array}{l} \dot{h_{1}}=h_{1}(\mu -b_{}p_{1}) \end{array}$$

where $\dot{h_{1}}=\frac{d\langle n_{H_{1}}\rangle }{\text{dt}N_{\text{avg}}}$ and the frequencies of *H*_*i*_ and *P*_*i*_ are given by *h*_*i*_ and *p*_*i*_. In the same way, we can derive deterministic differential equations for the frequencies of the other types,

(10) $$\begin{array}{l} \dot{h_{1}}=h_{1}(\mu -bp_{1})\; \end{array}$$

(11) $$\begin{array}{l} \dot{h_{2}}=h_{2}(\mu -bp_{2})\; \end{array}$$

(12) $$\begin{array}{l} \dot{p_{1}}=p_{1}(bh_{1}-c)\; \end{array}$$

(13) $$\begin{array}{l} \dot{p_{2}}=p_{2}(bh_{2}-c)\; \end{array}$$

### Stochastic simulations

The Gillespie algorithm gives an exact numerical solution of the Master equation of the system [41,70,71]. Our stochastic simulations are implementations of this algorithm with the transition rates as defined in Eqs. 6. Since the population size is not constrained, this simulation method includes a stochastic analogue of the Lotka-Volterra cycles.

We computationally remove the Lotka-Volterra cycles by culling the population of each species after *N*_avg_ transitions have taken place. During the *N*_avg_ transitions the types within a species can evolve to different frequencies. But in the end they are reset to sum up to *N*_avg_ while maintaining the relative abundances. The Gillespie method is discrete in the number of individuals but continuous in time. The unit of time is the same as in the deterministic system.

Alternatively we can consider a small amount *ε* of interactions between the otherwise independent Lotka-Volterra interactions. This is then represented by the following set of differential equations,

(14) $$\begin{array}{l} \dot{h_{1}}=h_{1}(\mu -bp_{1}-\varepsilon p_{2})\; \end{array}$$

(15) $$\begin{array}{l} \dot{h_{2}}=h_{2}(\mu -bp_{2}-\varepsilon p_{1})\; \end{array}$$

(16) $$\begin{array}{l} \dot{p_{1}}=p_{1}(bh_{1}+\varepsilon h_{2}-c)\; \end{array}$$

(17) $$\begin{array}{l} \dot{p_{2}}=p_{2}(\varepsilon h_{1}+bh_{2}-c).\; \end{array}$$

**Figure 5:**
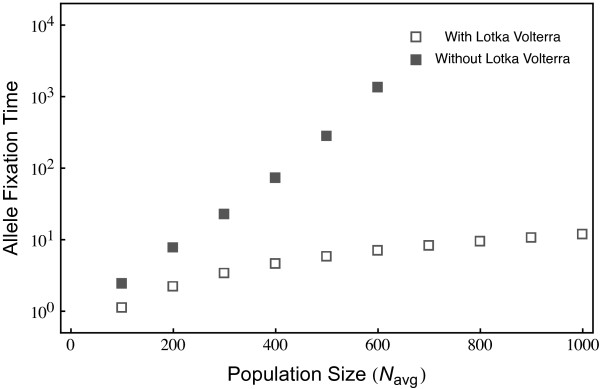
**Allele fixation/extinction times for any of the interacting types when we do include a slight interaction between the otherwise independent Lotka-Volterra cycles.** As compared to Figure 2 the fixation times in the case without Lotka-Volterra oscillations reduce with slight interaction between independent cycles. However for the case with Lotka-Volterra oscillations the fixation times are practically unchanged. For all simulations the initial condition were *H*_1_=*H*_2_=*N*_*avg*_/2, *P*_1_=90*N*_*avg*_/100, *P*_2_=10*N*_*avg*_/100, and the parameters *μ*=5, *c*=2.5, *b*=10/*N*_*avg*_ and *ε*=0.1*b* with averages over 10^6^ realizations).

Even for this case, including Lotka-Volterra interactions causes a faster extinction of the Red Queen cycles involving all four types. As an example we provide simulation results where in addition to similar parameters as in Figure 2 we add a *ε*=0.1*b* (Figure 5). Although the fixation time is elevated as compared to the case with no interactions (Figure 2), they are still not comparable to the extremely high fixations times observed when Lotka-Volterra dynamics is excluded.

## Competing interests

The authors declare that they have no competing interests.

## Authors’ contributions

HS and AP conceived the project. CSG and AT developed the model and performed simulations. All authors analysed the results and wrote the manuscript. All authors read and approved the final manuscript.
